# The Female Sexual Function Index: Transculturally Adaptation and Psychometric Validation in Spanish Women

**DOI:** 10.3390/ijerph17030994

**Published:** 2020-02-05

**Authors:** Beatriz Sánchez-Sánchez, Beatriz Navarro-Brazález, Beatriz Arranz-Martín, Óscar Sánchez-Méndez, Irene de la Rosa-Díaz, María Torres-Lacomba

**Affiliations:** Physiotherapy in Women’s Health (FPSM) Research Group, Physiotherapy Department, Faculty of Medicine and Health Sciences, University of Alcalá, Alcalá de Henares, 28805 Madrid, Spain

**Keywords:** FSFI, Spanish, pelvic floor disorders, sexual dysfunction, validity, reliability, responsiveness

## Abstract

Background: The evaluation of sexual function is an important outcome in women who suffer some pelvic floor disorders (PFD). The Female Sexual Function Index (FSFI) is the most widely used questionnaire to evaluate the sexual health in female population. This study presents the adaptation and psychometric validation of the FSFI for Spanish women with PFD. Methods: The Spanish version of the FSFI was developed through the forward and backward translation process. The psychometric properties of reliability, validity, responsiveness, and feasibility were conducted in Spanish women with PFD who were assigned to the case or control group (with or without sexual dysfunction respectively). Results: A total of 323 Spanish women with PFD were recruited. The cross-cultural adaptation of the Spanish FSFI achieved a good semantic, conceptual, idiomatic, and content equivalence. The test-retest reliability was shown to be high in all of the cases. The convergent validity showed high results in the domain intercorrelations between each domain and total FSFI. The discriminant validity showed statistically significant differences between sexual dysfunction and control groups. The responsiveness was shown to be moderate to good in the dimensions and excellent in the total FSFI. Conclusions: Spanish FSFI can be used as a reliable, valid, responsive, and feasible instrument for assessing sexual function in women.

## 1. Introduction

Sexual health is recognized by the World Health Organization as the complete well-being state related to sexuality, including physical, emotional, mental, and social welfare [1]. The compromise of sexual health supposes the development of sexual dysfunction, which affects nearly 40%–50% of women [2,3]. Different definitions of all forms of female sexual dysfunction (FSD) have been described. In 2015, the Fourth International Consultation on Sexual Medicine adopted the definitions of sexual dysfunction in women, consisting of: hypoactive sexual desire dysfunction, female sexual arousal dysfunction, female orgasmic dysfunction, female genital-pelvic pain dysfunction, persistent genital arousal disorder, postcoital syndrome, hypohedonic orgasm, and painful orgasm [4].

FSD has multifactorial causes, it is associated with a wide range of psychosocial and biomedical risk factors, like urogynaecological disorders or several metabolic diseases, such as diabetes mellitus, obesity, and metabolic syndrome [5]. Sexual dysfunction involves physical, social, and psychological dimensions, affecting women’s quality of life [6]. It is a frequent problem among the women population, especially in urogynaecological patients [7]. In this way, different studies have found that sexual complaints are common among women with pelvic floor disorders (PFD), recognizing the urinary incontinence, overactive bladder, or pelvic organ prolapse as risks factors for sexual dysfunction [8,9,10,11,12]. Twenty-five percent to 50% of women who seek treatment for PFD report an impairment in sexual function [9].

Thus, the evaluation of sexual function in women is an important outcome in different medical specialties and it is recognized as an integral part of quality of life. This evaluation is important, especially in women who suffer some PFD, since PFD symptoms are associated with sexual dysfunction, reduced sexual arousal, infrequent orgasm, and dyspareunia [13]. It is necessary to perform not only the clinical assessment in order to quantify, classify, and design an adequate treatment for PFD, but also the patients’ subjective perceptions. Some specific tools have been created to measure the subjective perception of quality of life and symptoms in women with PFD. The Pelvic Floor Distress Inventory short form and the Pelvic Floor Impact Questionnaire short form are psychometrically-validated and self-administered questionnaires that can address the presence and severity of PFD symptoms and their impact on quality of life [14]. The Pelvic Floor Distress Inventory short form and the Pelvic Floor Impact Questionnaire short form have been adapted and validated into Spanish language [15]. Although these questionnaires are recommended and widely used for PFD patients, it does not include questions regarding sexual function. Therefore, an assessment tool for female sexual function is necessary for PFD in women within the Spanish population. Nevertheless, the creation of new assessment tools is expensive and time consuming; for that, it is recommended to adapt and validate existing questionnaires in different populations where they will be administered. This way, the Female Sexual Function Index (FSFI) is the most widely used questionnaire for evaluating the sexual health in female population [16]. FSFI was developed by Rosen et al. in 2000, and it is a 19-item multidimensional self-reporting measure that quantified female sexual dysfunction in six domains: desire, arousal, lubrication, orgasm, satisfaction, and pain [17]. FSFI has been adapted and validated into more than 20 languages (e.g., [16,18,19,20,21]) and it is regarded as the de *facto* “gold standard” to assess the female sexual function [22]. Although there are two Spanish-Hispanic versions validated for Chilean and Colombian women [23,24], those versions could not be used in Spain, mainly because even if it is the same language, there are different expressions, contexts, and cultures and its use could imply an important penalization in the obtained data. Despite being a recommended international questionnaire, the Spanish version of FSFI in female Spanish sample has not been found. The aim of the present study has been adapting the original English FSFI into Spanish, and evaluating its psychometric properties of reliability, validity, responsiveness, and feasibility among Spanish women with PFD to considerate it as a reference instrument to assess female sexual function.

## 2. Materials and Methods

### 2.1. Translation and Cultural Adaptation

This study was a cross-sectional observational study that was conducted from March 2014 to February 2017. This study (OE21/2013) was approved by «BLINDED» Hospital’s Clinical Research Ethics Committee in «BLINDED».

The study was divided into three phases pursuant to the International Society for Pharmacoeconomics and Outcomes Research (ISPOR) Task Force for Translation and Cultural Adaptation [25]. Two English-Spanish translators (native Spanish speakers) translated the original version of the FSFI, written in English, to create equivalent texts regarding semantics, concepts, idiomatic and contents. Both of the translators worked separately and obtained two versions in Spanish that proved to be equivalent to the original version. The translators proofread the translations and, together with the research team, agreed to the synthesis of the Spanish translation. 

Secondly, two professional Spanish-English translators (native English speakers) worked autonomously to get the back-translation, thus creating two English versions from the previous Spanish translation. Subsequently, an Expert Committee, considering both the back-translation and the Spanish-translated text, agreed to the Spanish version of the FSFI and to their equivalence to the original.

Thirdly, the Spanish preliminary version was given to 20 native Spanish-speaking women who fulfilled the inclusion criteria, for them to test its feasibility, equivalence, and comprehensibility. They filled in the questionnaire and were later interviewed in person so that any difficulties to understand the items could be identified and corrected. The FSFI’s Spanish version was obtained after identifying and amending some minor details. The study reporting followed the “Strengthening the Reporting of Observational studies in Epidemiology” (STROBE) guidelines (Table S1 Supplementary Material).

### 2.2. Participants and Procedure

All women who were assessed by the «BLINDED» Research Group of the «BLINDED» and fulfilled the inclusion criteria were informed about the study and invited to participate. So, the written informed consent was obtained from all of the participants. The inclusion criteria were the following: female diagnosed with PFD, above 18 years of age, having been sexually active within the previous four weeks, being able to read and to understand Spanish language. The exclusion criteria were: current pregnancy or mental incapacity to fill in the questionnaire. The recruited women were provided with a brief study explanation by a researcher and diagnosed of FSD based on the Diagnostic and Statistical Manual of Mental Disorders, Fifth Edition (DSM-5) [26] that assessed the presence (or absence) of FSD, and that was administered during a face-to-face interview. In this way, with this diagnosis, the recruited women were assigned to the case group (with FSD) or to the control group (without FSD). The sample size has been based on the general recommendations of Terwee et al. [27], which recommend a subject-to-item ratio of at least 4:1, with a minimum of 100 subjects.

At baseline, women completed the Spanish versions of FSFI and a sociodemographic form. A subsample of 31 women filled in the FSFI again two-four weeks later in order to analyze the test-retest reliability. No treatment was delivered during this time; this interval was chosen to ensure that women’s symptoms remained unchanged and long enough to ensure that they would not recall their baseline responses. A subgroup of women from the FSD group was evaluated again after the Physiotherapy treatment to evaluate responsiveness. The average time was recorded.

### 2.3. Measures

The tools used in this study included: participant characteristics’ form, DSM-5, and the Spanish versions of FSFI. 

#### 2.3.1. Participant Characteristics

The women’s baseline characteristics collected were: age, educational level, marital status, occupational status, annual family income, vaginal delivery, and menopause 

#### 2.3.2. Diagnostic and Statistical Manual of Mental Disorders, Fifth Edition

The DSM-5 is a widely psychiatric system used internationally for classifying the sexual dysfunction proposed by the American Psychiatric Association [26].

#### 2.3.3. Female Sexual Function Index

The FSFI is a multidimensional self-report instrument that was developed by Rosen et al. to assessing female sexual function over the past 4 weeks [17]. It contains 19 items into six subscales: sexual desire (items 1 and 2), arousal (items 3, 4, 5 and 6), lubrication (items 7, 8, 9, and 10), orgasm (items 11, 12, and 13), satisfaction (items 14, 15 and 16), and pain (items 17, 18, and 19). The score range for items 3 to 14 and 17 to 19 between 0–5; for items 1, 2, 15, and 16 between 1–5. The full-scale score range is from 2 to 36, with the higher scores indicating better sexual function.

### 2.4. Data Analysis

The FSFI’s Spanish version was tested for reliability, validity, responsiveness, and feasibility.

Statistical analysis of data that were obtained was conducted while using SPSS version 24.0. Statistical significance was assumed at *p* < 0.05. COSMIN recommendations were used as a guide for evaluating the psychometric properties [28]. 

Reliability was assessed by the test-retest reliability and internal consistency. The test-retest reliability (the degree to which a measurement is free from error) [28] was measured by the intraclass correlation coefficient (ICC) for the total score and scores of each domain. Good values of test-retest reliability are considered greater than 0.7 [27]. A test-retest analysis of two-four weeks was used. Internal consistency (the degree of interrelatedness among each domain in the questionnaire and for all the items) was measured by means of Cronbach’s alpha (α), considered a value of 0.7 good reliability, the higher the value, the greater the internal consistency [27,29]. 

Validity was assessed by the content and construct validity (factorial, convergent, and discriminant). Although content validity for assessing the ability of items to collect health status was guaranteed by the validation of the original scale, in this study, the Expert Committee’s (consisting of a methodologist, three health professionals (one physical therapist, one physician, one gynecologist), one language professional, and two translators (forward and backward translators)) opinion was also taken into account to judge the ability of questionnaires to assess all dimensions, and the pilot study subjects opinion of 20 women, who reported completing the questionnaires.

Factorial validity, the degree to which each item relates to the hypothesized domain with which it is associated was conducted [17]. Factor structure was assessed with principal component analysis and a subsequent confirmatory factor analysis. A varimax rotation with Kaiser normalization was used in the total sample (N = 323) at baseline to evaluate the principal components or factors. It was considered a Kaiser-Meyer-Olkin (KMO) >0.80 optimal and <0.5 insufficient [16]. The Bartlett test of sphericity was calculated to ascertain the correlations between the variables and the appropriateness of the factor model. 

The convergent construct validity is the extent to which scores demonstrate expected logical relations with other variables. It was measured with the FSFI domain intercorrelations and the correlations between each domain and total score. While using the Pearson correlation, a value of <0.3 was considered to indicate a weak correlation, one of 0.3 to 0.5 a moderate correlation, and one of >0.5 a strong correlation [30].

The discriminant construct validity was calculated with an independent Student’s *T*-test while comparing the difference of the scores of FSFI domains between case and control group.

The responsiveness or sensitivity to change was assessed in a subgroup of women with FSD who carried out a Physiotherapy treatment for pelvic floor during eight weeks. These women filled in the FSFI twice: at baseline, and after the Physiotherapy intervention. Three distribution-based methods were used in order to evaluate responsiveness: the p values generated using the Wilcoxon signed-rank test, the effect size (ES), and the standardized response means for the change (SRM) in scores from pre- and post-Physiotherapy intervention using the paired t test. For SRM and ES, a value of 0.2–0.5 was considered as small, of 0.5–0.8 as moderate, of above 0.8–1.0 as good, and of more than 1.0, excellent [31,32,33].

The percentage of unanswered individual items and the percentage of women who did not answer any of the items were analyzed to evaluate FSFI’s Spanish version feasibility. Additionally, the average administration time was calculated. 

## 3. Results

### 3.1. Study Participants Characteristics

Three hundred and twenty-three consecutive women were recruited from March 2014 to May 2018; the sample characteristics are presented in Table 1. The sample women were categorized into the FSD group (167 (51.7%)) and control group (156 (48.3%)) according to the DSM-5 criteria. 

### 3.2. Cultural Adaptation 

The cross-cultural adaptation of FSFI’ Spanish version achieved a good semantic, conceptual, idiomatic, and content equivalences.

In the pilot study, most of the items were well understood. During the translation process and cognitive interviews, some words were identified and discussed until the most appropriate wording was found, i.e., “vaginal intercourse”, “sexual intercourse”, “intercourse”, and “vaginal penetration” were translated like “coito vaginal”. After having been revised by the expert panel, the Spanish final version was obtained (Table S2 Supplementary Material). This Spanish final version contained 19 questionnaire items that were divided into six dimensions, with a comparable structure to the original English version.

### 3.3. Reliability

31 women (16 from FSD group and 15 from control group) completed the FSFI questionnaire twice within 2–4 weeks from the basal visit in order to evaluate the test-retest reliability. The test-retest reliability showed high in all the cases, with ICC from 0.90 (95% CI: 0.792–0.959) to 0.94 (95% CI: 0.872–0.975) in the dimensions and with 0.96 (95% CI: 0.910–0.982) in the total score (Table 2).

In terms of internal consistency, FSFI showed high Cronbach’s α coefficient values in all dimensions for all groups, both in the different dimensions the total score (Table 3).

### 3.4. Validity

In the pilot study, the revisions of women and Expert Committee’ guaranteed adequate content validity of the Spanish version of FSFI, enough to evaluate the female sexual function.

Regarding the factor structure of the Spanish FSFI, a value of 0.861 was obtained in the Kaiser-Meyer-Olkin, with a Bartlett sphericity test. First, we used a minimum eigenvalue of 1.0 as a criterion factor extraction, and then four factors were identified with minimum eigenvalue of 1.30 and a total of 73.94% of the variance: desire/arousal, lubrication, orgasm/satisfaction, and pain. A second principal component analysis was calculated based in the studies of the original FSFI and of the FSFI confirmatory factor analysis [34] that decided a six-factor structure. In this way, the six-factor solution accounted a total of 82.57% of the variance and the lowest eigenvalue was 0.76, including the domains of: desire, arousal, lubrication, orgasm, satisfaction, and pain (Table 4). In this six-factor analysis, the minimum factor loading was 0.633. 

Convergent construct validity, calculated with the domain intercorrelations and correlations between each domain and total FSFI, using Pearson’s, showed high in all of the groups and observed higher values in the total group. The values were higher in the correlation of total FSFI and domains than in interdomains. The domains that showed higher intercorrelations were observed for arousal with desire, orgasm, and satisfaction; and, orgasm with satisfaction (Table 5). 

About the discriminant construct validity, the scores for each FSFI domains and total FSFI showed statistically significant differences between the FSD group and the control group (*p* < 0.05) (Table 6). The difference between the total scale score was 5.128 (95% CI: 4.087–6.170).

### 3.5. Responsiveness

To assess responsiveness, a total of 92 women were recruited from the FSD group. Table 7 presents the baseline characteristics of these women. About the ES and SRM, they showed moderate to good in the FSFI dimensions, the lowest value being in the desire dimension (ES 0.69 and SRM 0.58; *p* < 0.001) and the highest in the pain dimension (ES 1.05 and SRM 1.01; *p* < 0.001). Regarding the ES and SRM of the total FSFI, both of the values showed excellent responsiveness (ES 1.24 and SRM 1.11; *p* < 0.001) (Table 8).

### 3.6. Feasibility

Concerning feasibility, the average time for questionnaire administration was 4.33 (1.74) min. for the FSFI’s Spanish version. All of the FSFI questionnaires were self-reported by the women in the consult. Accordingly, the women with non-response item were zero in the six dimensions, and the majority of items were easily understood. 

## 4. Discussion

PFD have been shown to have a significant impact on quality of life, which includes the sexual function. The FSFI is regarded as the gold standard to evaluate female sexual function [22]. It was developed and validated in English language and it has been adapted and validated to numerous languages, which allows for the possibility of international multicenter studies and comparing results of studies from different countries. In this way, the Chilean and Colombian FSFI [23,24] must not be used in Spanish women from Spain, because they have been cross-cultural adapted and validated in a different sociocultural environment. In fact, some of the authors have proposed Guidelines in order to adapt questionnaires into the same language within different cultures [35,36], so it has been recognized that culture affects sexual function [37], and it is usually modulated by moral, religious, or ethical criteria, so the results of research in a country may not be applicable in another. Secondly, it is essential to assess the psychometric properties of the validated tool to the target population, in our case, Spanish women. Therefore, using a validated tool for Iberoamerican countries in a Spanish population might be a significant source of bias in the obtained data.

The method used in the present study for the FSFI’s Spanish version is similar to that performed in the others FSFI’s validity versions, including a first stage of translation of original English FSFI to Spanish FSFI questionnaire and another to evaluate the psychometric properties in a female sample. 

As we know, this study is the first FSFI validity study that evaluates responsiveness. It is widely argued that outcome measures in clinical trials should show not only validity and reliability, but also responsiveness or sensitiveness to change [38]. Responsiveness is the ability to detect changes that occur as a result of therapy or disease progression and it has been suggested as one criterion to choose among the scales used to evaluate the efficacy of a therapeutic intervention. In fact, a low response can produce the lack of difference when a real difference, in fact, exists (risk of a type II error), which can lead to an underestimation on the effect of treatment [39]. Hence, we consider responsiveness to be a fundamental psychometric characteristic that should be assessed in our study. Regarding the female sample, we have included women with PFD, which differs from women included in others FSFI validations. Sociodemographic characteristics showed that the age of sample was similar to the original and validations FSFI studies, in our study of 48 [8] in the FSD group and of 40 [11] in the control group. In the Spanish validation, like in the rest of them, the age of the FSD group was higher than that of the control group; in fact, the age has been identified as a significant factor that is predictive of the worsening of the sexual function [11]. 

The results concerning reliability, internal consistency, and test-retest reproducibility showed high. Cronbach’s Alpha showed similar in all the groups, with good values in all of the cases. The test-retest reliability demonstrated significant correlations two to four weeks, with Pearson correlation coefficient of, at least, 0.90. 

Convergent construct validity showed that all of the domains were correlated in the total group. The highest correlations were found between arousal with desire, orgasm, and satisfaction and between orgasm with satisfaction. These results can be also observed in the original study. The domains correlations were higher for the total group, it was also found in the original and Malay versions [17,40].

In the present study, analysis of discriminant validity has been measured calculating the difference between the FSD and control group. This method has also been adopted in others validations, like original, the Chinese, the Iranian, and the Malay [16,17,19,40], and, as in these studies, our results showed that the scores for each domains and full-scale of FSFI are significantly higher for the FSD group as compared to control group.

Regarding the principal component analysis in the present study, like in the Chinese validation, a first principal component analysis identified four factors, but eventually six factors were identified based in the FSFI original structure and in the FSFI confirmatory factor analysis study. The first four-factor solution accounted the 73.94% of the explained variance, with a lowest eigenvalue of 1.30 and including the domains of desire/arousal, lubrication, orgasm/satisfaction, and pain. In other way, the six-factor solution every extracted factor had a reasonable and clear explanation that accounted for the 82.57% of the explained variance and showed high-factor loading for the six identified dimensions: desire, arousal, lubrication, orgasm, satisfaction, and pain, with a minimum loading of 0.633 and a maximum loading of 0.948, showed a reasonable and clear explanation of the extracted factor and therefore, the factorial validity of the Spanish FSFI. Previous FSFI validations showed inconsistent findings regarding factor solution, identifying from three (Taiwan version [41]) to six factors, although most studies have finally showed six factors, like the original study did (Chinese, Arabic, and Colombian versions).

As a limitation in this study, we find that the sample are women with PFD, which could influence in the female sexual health and, so, it could affect the obtained data. This fact might involve a limitation in the generalizability of the application of FSFI to the general Spanish women population. In this way, future validation in diverse subpopulation are recommended. 

## 5. Conclusions

The Spanish FSFI can be used as a reliable, valid, responsive, and feasible instrument for assessing sexual function in Spanish women with PFD.

## Figures and Tables

**Table 1:** Baseline characteristics of participants.

	FSD Group (N = 167)	Control Group (N = 156)
Age (years, X (SD))	48 (8)	40 (11)
Education level (*n* (%))		
Primary School	43 (25.7%)	22 (14.1%)
High School	45 (26.9%)	40 (25.6%)
College or University	79 (47.3%)	94 (60.2%)
Marital Status (*n* (%))		
Married	116 (69.4%)	86 (55.1%)
In couple	48 (28.7%)	57 (36.5%)
Single	3 (1.7%)	13 (8.3%)
Occupational status (*n* (%))		
Housewife	30 (17.9%)	13 (8.3%)
Employee	123 (73.6%)	120 (76.9%)
Unemployed	11 (6.5%)	7 (4.4%)
Student	3 (1.7%)	16 (10.2%)
Annual family income (€) (*n* (%))		
<12.000 €	16 (9.5%)	11 (7 %)
12.000–24.000 €	35 (20.9%)	34 (21.7%)
>24.000–36.000 €	57 (34.1%)	44 (28.2%)
>36.000–48.000 €	16 (9.5%)	23 (14.7%)
>48.000 €	14 (8.3%)	3 (1.7%)
Prefer don’t say it	29 (17.4%)	40 (25.6%)
Vaginal delivery (*n* (%))		
Nulliparous	41 (24.5%)	62 (39.7%)
1	57 (34.1%)	46 (29.4%)
2	58 (34.7%)	35 (22.4%)
3	6 (3.5%)	12 (7.6%)
4 or more	5 (2.9%)	1 (0.6%)
Menopause (*n* (%))		
Yes	58 (34.7%)	35 (22.4%)
No	109 (65.2%)	121 (77.6%)

*X* (SD): Mean (Standard Deviation); FSD: Female Sexual Dysfunction.

**Table 2:** Test-retest reliability for dimensions and total Female Sexual Function Index (FSFI) (N = 31).

	ICC	95% CI
Desire	0.943	0.872–0.975
Arousal	0.907	0.792–0.959
Lubrication	0.939	0.864–0.973
Orgasm	0.916	0.811–0.963
Satisfaction	0.931	0.845–0.969
Pain	0.930	0.844–0.969
Total FSFI	0.960	0.910–0.982

ICC: interclass correlation coefficients. CI: confidence interval.

**Table 3:** Internal consistency reliability for dimensions and total FSFI (Cronbach’s Alpha).

	FSD Group (N = 167)	Control Group (N = 156)	Total Group (N = 323)
Desire	0.752	0.749	0.760
Arousal	0.723	0.749	0.745
Lubrication	0.739	0.763	0.756
Orgasm	0.741	0.747	0.753
Satisfaction	0.739	0.739	0.753
Pain	0.753	0.762	0.765
Total FSFI	0.793	0.837	0.850

FSD: Female Sexual Dysfunction.

**Table 4:** Factor analysis of the FSFI: factorial validity (N = 323).

	Factors					
	F1	F2	F3	F4	F5	F6
1. Desire: frequency	0.302	0.238	0.177	0.082	0.142	0.824
2. Desire: level	0.301	0.177	0.186	0.163	0.098	0.833
3. Arousal: frequency	0.707	0.173	0.209	0.084	0.239	0.419
4. Arousal: level	0.673	0.180	0.311	0.174	0.179	0.431
5. Arousal: confidence	0.717	0.200	0.162	0.211	0.345	0.239
6. Arousal: satisfaction	0.723	0.181	0.313	0.124	0.379	0.181
7. Lubrication: frequency	0.519	0.701	0.076	0.135	−0.019	0.113
8. Lubrication: difficulty	0.023	0.857	0.112	0.163	0.144	0.223
9. Lubrication: frequency of maintaining	0.359	0.767	0.196	0.201	0.030	0.081
10. Lubrication: difficulty in maintaining	0.028	0.846	0.192	0.114	0.256	0.139
11. Orgasm: frequency	0.460	0.045	0.404	−0.055	0.633	0.051
12. Orgasm: difficulty	0.194	0.170	0.185	0.146	0.822	0.188
13. Orgasm: satisfaction	0.308	0.176	0.384	0.040	0.702	0.076
14. Satisfaction: with closeness with partner	0.195	0.221	0.831	0.114	0.136	0.134
15. Satisfaction: with sexual relationship	0.144	0.111	0.850	0.123	0.263	0.143
16. Satisfaction: with overall sex life	0.334	0.193	0.676	0.008	0.344	0.202
17. Pain: frequency during vaginal penetration	0.041	0.249	0.020	0.635	0.278	0.344
18. Pain: frequency following vaginal penetration	0.123	0.191	0.087	0.948	0.097	0.154
19. Pain: level during or following vaginal penetration	0.143	0.095	0.103	0.886	−0.083	−0.058
Eigenvalue	9.7	2.23	1.45	1.30	0.87	0.76
% of explained variance	47.73%	11.74%	7.65%	6.82%	4.60%	4.03%

Principal component analysis using rotation method varimax with Kaiser normalization. Items with the highest factor loading in each principal component are shaded. F1: Arousal, F2: Lubrication, F3: Satisfaction, F4: Pain, F5: Orgasm, F6: Desire.

**Table 5:** FSFI Domains intercorrelations (Pearson r).

TOTAL GROUP	Desire	Arousal	Lubrication	Orgasm	Satisfaction	Pain	Total FSFI
Desire	1						
Arousal	0.676 (**)	1					
Lubrication	0.455 (**)	0.560 (**)	1				
Orgasm	0.439 (**)	0.685 (**)	0.427 (**)	1			
Satisfaction	0.464 (**)	0.632 (**)	0.467 (**)	0.694 (**)	1		
Pain	0.351 (**)	0.381 (**)	0.462 (**)	0.332 (**)	0.363 (**)	1	
Total FSFI	0.730 (**)	0.853 (**)	0.742 (**)	0.780 (**)	0.792 (**)	0.653 (**)	1
**FSD GROUP**	Desire	Arousal	Lubrication	Orgasm	Satisfaction	Pain	Total FSFI
Desire	1						
Arousal	0.614 (**)	1					
Lubrication	0.364 (**)	0.494 (**)	1				
Orgasm	0.275 (**)	0.638 (**)	0.307 (**)	1			
Satisfaction	0.370 (**)	0.619 (**)	0.352 (**)	0.656 (**)	1		
Pain	0.210 (**)	0.273 (**)	0.439 (**)	0.180 (*)	0.214 (**)	1	
Total FSFI	0.640 (**)	0.839 (**)	0.705 (**)	0.712 (**)	0.744 (**)	0.591 (**)	1
**CONTROL GROUP**	Desire	Arousal	Lubrication	Orgasm	Satisfaction	Pain	Total FSFI
Desire	1						
Arousal	0.644 (**)	1					
Lubrication	0.358 (**)	0.459 (**)	1				
Orgasm	0.466 (**)	0.616 (**)	0.389 (**)	1			
Satisfaction	0.419 (**)	0.519 (**)	0.498 (**)	0.655 (**)	1		
Pain	0.386 (**)	0.376 (**)	0.259 (**)	0.418 (**)	0.483 (**)	1	
Total FSFI	0.731 (**)	0.798 (**)	0.657 (**)	0.792 (**)	0.814 (**)	0.650 (**)	1

** *p* < 0.01 is considered statistically significant. FSD: Female Sexual Dysfunction.

**Table 6:** Discriminant construct validity: correlation of FSFI domains scores between cases and control group (Paired *t*-test).

	FSD Group (N = 167) *X* (SD)	Control Group (N = 156) *X* (SD)	Mean Difference	95% CI	T Value	*p* Value
Desire	3.3 (1.12)	4.2 (0.95)	0.899	0.672–1.126	7.797	<0.001
Arousal	4.2 (1.26)	5.1 (0.80)	0.890	0.661–1.119	7.642	<0.001
Lubrication	4.5 (1.33)	5.4 (0.82)	0.925	0.685–1.166	7.566	<0.001
Orgasm	4.4 (1.26)	5.3 (0.85)	0.891	0.657–1.125	7.499	<0.001
Satisfaction	4.5 (1.18)	5.2 (0.95)	0.721	0.487–0.955	6.063	<0.001
Pain	4.8 (1.48)	5.6 (0.88)	0.792	0.527–1.057	5.880	<0.001
Total FSFI	25.7 (5.49)	30.9 (3.99)	5.128	4.087–6.170	9.691	<0.001

*p* < 0.05 is considered statistically significant. *X* (SD): Mean (Standard Deviation); FSD: Female Sexual Dysfunction. CI: confidence interval.

**Table 7:** Baseline characteristics of women for responsiveness (N = 92).

Age (years, *X* (SD))	50 (6)
Education level (*n* (%))	
Primary School	10 (10.8%)
High School	24 (26.0%)
College or University	58 (63.0%)
Marital Status (*n* (%))	
Married	49 (53.3%)
In couple	43 (46.7%)
Single	-
Occupational status (*n* (%))	
Housewife	11 (11.9%)
Employee	76 (82.6%)
Unemployed	5 (5.4%)
Student	-
Annual family income (€) (*n* (%))	
<12.000 €	4 (4.3%)
12.000–24.000 €	29 (31.5%)
>24.000–36.000 €	34 (36.9%)
>36.000–48.000 €	15 (16.3%)
>48.000 €	4 (4.3%)
Prefer don’t say it	6 (6.5%)
Vaginal delivery (*n* (%))	
Nulliparous	2 (2.2%)
1	63 (68.5%)
2	27 (29.3%)
3	-
4 or more	-
Menopause (*n* (%))	
Yes	33 (64.1%)
No	59 (35.9%)

*X* (SD): Mean (Standard Deviation); FSD: Female Sexual Dysfunction.

**Table 8:** FSFI Mean change in scores, and responsiveness.

N = 92	Pretreatment *X* (SD) Score	Posttreatment *X* (SD) Score	Mean Change in Score (SD)	95% CI	T Value	*p* Value	Effect Size (ES)	Standardized Response Mean (SRM)
Desire	3.1 (1.01)	3.8 (0.99)	0.7 (1.21)	0.492–0.995	5.873	<0.001	0.69	0.58
Arousal	3.4 (1.31)	4.7 (1.09)	1.3 (1.58)	0.977–1.631	7.914	<0.001	0.99	0.82
Lubrication	3.4 (1.63)	4.9 (1.25)	1.5 (1.76)	1.149–1.882	8.221	<0.001	0.92	0.85
Orgasm	3.5 (1.65)	5.0 (1.17)	1.5 (1.79)	1.197–1.941	8.378	<0.001	0.91	0.84
Satisfaction	3.8 (1.33)	5.0 (1.02)	1.2 (1.46)	0.952–1.560	8.212	<0.001	0.90	0.82
Pain	3.4 (1.81)	5.3 (1.01)	1.9 (1.89)	1.550–2.336	9.821	<0.001	1.05	1.01
Total FSFI	20.7 (6.71)	29.0 (5.26)	8.3 (7.45)	6.788–9.878	10.715	<0.001	1.24	1.11

*p* < 0.05 is considered statistically significant. *X* (SD): Mean (Standard Deviation); SD: standard deviation; ES effect size; SRM: standardized response means. CI: confidence interval.

## Supplementary Materials

The following are available online at https://www.mdpi.com/1660-4601/17/3/994/s1, Table S1: STROBE Statement, Table S2: Spanish FSFI (*Índice de la Función Sexual Femenina*) and scoring.
