# Erratum: Sánchez-Sánchez, B., et al. The Female Sexual Function Index: Transculturally Adaptation and Psychometric Validation in Spanish Women. *Int. J. Environ. Res. Public Health* 2020, *17*(3), 994

**DOI:** 10.3390/ijerph17124355

**Published:** 2020-06-17

**Authors:** Beatriz Sánchez-Sánchez, Beatriz Navarro-Brazález, Beatriz Arranz-Martín, Óscar Sánchez-Méndez, Irene de la Rosa-Díaz, María Torres-Lacomba

**Affiliations:** Physiotherapy in Women’s Health (FPSM) Research Group, Physiotherapy Department, Faculty of Medicine and Health Sciences, University of Alcalá, Alcalá de Henares, 28805 Madrid, Spain; beatriz.sanchez@uah.es (B.S.-S.); b.navarro@uah.es (B.N.-B.); beatriz.arranz@edu.uah.es (B.A.-M.); osmfisio@gmail.com (Ó.S.-M.); maria.torres@uah.es (M.T.-L.)

The authors wish to make the following correction to their paper published in the *International Journal of Environmental Research and Public Health* [1]:

## 1. Change in Main Body Paragraphs

There are two mistakes in this article [1]: 

On page 2, in the first paragraph of: 2.1. Translational and Cultural Adaptation subsection (2. Materials and Methods section), the sentence “This study (OE21/2013) was approved by «BLINDED» Hospital’s Clinical Research Ethics Committee in «BLINDED».”.

Will be corrected to: “This study (OE21/2013) was approved by Príncipe de Asturias Hospital’s Clinical Research Ethics Committee in Alcalá de Henares (Madrid).”. 

On page 3, in the first paragraph of: 2.2. Participants and Procedure subsection (2. Materials and Methods section), the sentence “All women who were assessed by the «BLINDED» Research Group of the «BLINDED» and …”.

Will be corrected to: “All women who were assessed by Physiotherapy in Women’s Health Research Group of Alcalá University (Madrid, Spain) and …”.

## 2. References

There is one mistake:

On page 13, Reference number 33: “Sánchez, B.S.; Lacomba, M.T.; Brazález, B.N.; Téllez, E.C.; Da Costa, S.P.; Ortega, C.G. Responsiveness of the Spanish Pelvic Floor Distress Inventory and Pelvic Floor Impact Questionnaires Short Forms (PFDI-20 and PFIQ-7) in women with pelvic floor disorders. *Eur. J. Obstet. Gynecol. Reprod. Biol*. **2015**, *190*, 20–25.”.

Will be corrected to: “Sánchez-Sánchez, B.; Torres-Lacomba, M.; Navarro-Brazález, B.; Cerezo-Téllez, E.; Pacheco-Da-Costa, S.; Gutiérrez-Ortega, C. Responsiveness of the Spanish Pelvic Floor Distress Inventory and Pelvic Floor Impact Questionnaires Short Forms (PFDI-20 and PFIQ-7) in women with pelvic floor disorders. *Eur. J. Obstet. Gynecol. Reprod. Biol*. **2015**, *190*, 20–25.”.

The authors would like to apologize for any inconvenience caused to the readers by these changes.
