# Supplementary material for: The Female Sexual Function Index: Transculturally Adaptation and Psychometric Validation in Spanish Women
Source: Int J Environ Res Public Health. 2020 Feb 5;17(3):994. doi: 10.3390/ijerph17030994 (PMC7037847; doi:10.3390/ijerph17030994)
Supplement: Supplementary file 1 [file ijerph-17-00994-s001.zip › ijerph-697101-SI/Supplementary material/SUPPLEMENTARY MATERIAL_S1_SPANISH FSFI.pdf]

SUPPLEMENTARY MATERIAL

ÍNDICE DE LA FUNCIÓN SEXUAL FEMENINA

Las siguientes preguntas son sobre sus sentimientos y respuestas sexuales durante las últimas 4 semanas. Por favor, conteste a las siguientes preguntas lo más honesta y claramente posible. Sus respuestas serán completamente confidenciales. Definiciones:

- o *Actividad sexual*: incluye caricias, juegos sexuales, masturbación y coito vaginal
- o *Coito vaginal:* se define como la introducción del pene en la vagina
- o *Estimulación sexual*: incluye juegos sexuales con la pareja, auto estimulación (masturbación) o fantasías sexuales.

MARQUE UNA SOLA RESPUESTA A CADA PREGUNTA:

Deseo o interés sexual: es la sensación que incluye el deseo de tener una experiencia sexual, sentirse receptiva a la iniciación sexual de la pareja y pensar o fantasear sobre tener sexo.

1. **En las últimas 4 semanas, ¿con qué frecuencia experimentó deseo o interés sexual?**
   - € Casi siempre o siempre
   - € La mayoría de las veces (más de la mitad de las veces)
   - € A veces (aproximadamente la mitad de las veces)
   - € Pocas veces (menos de la mitad de las veces)
   - € Casi nunca o nunca

2. **En las últimas 4 semanas, ¿cómo calificaría su nivel (grado) de deseo o interés sexual?**
   - € Muy alto
   - € Alto
   - € Moderado
   - € Bajo
   - € Muy bajo o nada

Excitación sexual: es la sensación que incluye aspectos físicos y mentales de la exaltación sexual. Puede incluir sensación de calor o latidos en los genitales, lubricación (humedad) o contracciones musculares.

3. **En las últimas 4 semanas, ¿con qué frecuencia sintió excitación sexual durante la actividad sexual o coito vaginal?**
   - € Sin actividad sexual
   - € Casi siempre o siempre
   - € La mayoría de las veces (más de la mitad de las veces)
   - € A veces (aproximadamente la mitad de las veces)
   - € Pocas veces (menos de la mitad de las veces)
   - € Casi nunca o nunca
4. **En las últimas 4 semanas, ¿cómo calificaría su nivel de excitación sexual durante la actividad sexual o coito vaginal?**
   - € Sin actividad sexual
   - € Muy alto
   - € Alto
   - € Moderado
   - € Bajo
   - € Muy bajo o nada

5. **En las últimas 4 semanas, ¿Cuánta confianza tuvo para conseguir excitarse durante la actividad sexual o coito vaginal?**
   - € Sin actividad sexual
   - € Confianza muy alta
   - € Confianza alta
   - € Confianza moderada
   - € Confianza baja
   - € Confianza muy baja o nada

6. **En las últimas 4 semanas, ¿con qué frecuencia se sintió satisfecha con su excitación durante la actividad sexual o coito vaginal?**
   - € Sin actividad sexual
   - € Casi siempre o siempre
   - € La mayoría de las veces (más de la mitad de las veces)
   - € A veces (aproximadamente la mitad de las veces)
   - € Pocas veces (menos de la mitad de las veces)
   - € Casi nunca o nunca

7. **En las últimas 4 semanas, ¿con qué frecuencia consiguió la lubricación vaginal (humedad vaginal) durante la actividad sexual o coito vaginal?**
   - € Sin actividad sexual
   - € Casi siempre o siempre
   - € La mayoría de las veces (más de la mitad de las veces)
   - € A veces (aproximadamente la mitad de las veces)
   - € Pocas veces (menos de la mitad de las veces)
   - € Casi nunca o nunca

8. **En las últimas 4 semanas, ¿cuánta dificultad encontró para lubricarse (humedad vaginal) durante la actividad sexual o coito vaginal?**
   - € Sin actividad sexual
   - € Extremadamente difícil o imposible
   - € Muy difícil
   - € Difícil
   - € Un poco difícil
   - € Sin dificultad

9. **En las últimas 4 semanas, ¿con qué frecuencia mantuvo la lubricación vaginal (humedad vaginal) hasta finalizar la actividad sexual o coito vaginal?**
   - € Sin actividad sexual
   - € Casi siempre o siempre
   - € La mayoría de las veces (más de la mitad de las veces)
   - € A veces (aproximadamente la mitad de las veces)
   - € Pocas veces (menos de la mitad de las veces)
   - € Casi nunca o nunca

10. En las últimas 4 semanas, ¿cuánta dificultad encontró para mantener la lubricación vaginal (humedad vaginal) hasta finalizar la actividad sexual o coito vaginal?

- € Sin actividad sexual
- € Extremadamente difícil o imposible
- € Muy difícil
- € Difícil
- € Un poco difícil
- € Sin dificultad

11. En las últimas 4 semanas, cuando tuvo una estimulación sexual o coito vaginal, ¿con qué frecuencia consiguió el orgasmo (clímax)?

- € Sin actividad sexual
- € Casi siempre o siempre
- € La mayoría de las veces (más de la mitad de las veces)
- € A veces (aproximadamente la mitad de las veces)
- € Pocas veces (menos de la mitad de las veces)
- € Casi nunca o nunca

12. En las últimas 4 semanas, cuando tuvo una estimulación sexual o coito vaginal, ¿cuánta dificultad tuvo para alcanzar el orgasmo (clímax)?

- € Sin actividad sexual
- € Extremadamente difícil o imposible
- € Muy difícil
- € Difícil
- € Un poco difícil
- € Sin dificultad

13. En las últimas 4 semanas, ¿cuánta satisfacción sintió con su capacidad para alcanzar el orgasmo (clímax) durante la actividad sexual o coito vaginal??

- € Sin actividad sexual
- € Muy satisfecha
- € Moderadamente satisfecha
- € Ni satisfecha ni insatisfecha
- € Moderadamente insatisfecha
- € Muy insatisfecha

14. En las últimas 4 semanas, ¿cuánta satisfacción sintió con el acercamiento emocional con su pareja durante la actividad sexual?

- € Sin actividad sexual
- € Muy satisfecha
- € Moderadamente satisfecha
- € Ni satisfecha ni insatisfecha
- € Moderadamente insatisfecha
- € Muy insatisfecha

15. **En las últimas 4 semanas, ¿cuánta satisfacción sintió con la relación sexual con su pareja?**
    - € Muy satisfecha
    - € Moderadamente satisfecha
    - € Ni satisfecha ni insatisfecha
    - € Moderadamente insatisfecha
    - € Muy insatisfecha

16. **En las últimas 4 semanas, ¿cuánta satisfacción sintió con su vida sexual en general?**
    - € Muy satisfecha
    - € Moderadamente satisfecha
    - € Ni satisfecha ni insatisfecha
    - € Moderadamente insatisfecha
    - € Muy insatisfecha

17. **En las últimas 4 semanas, ¿con cuánta frecuencia sintió molestias o dolor durante coito vaginal?**
    - € Sin coito vaginal
    - € Casi siempre o siempre
    - € La mayoría de las veces (más de la mitad de las veces)
    - € A veces (aproximadamente la mitad de las veces)
    - € Pocas veces (menos de la mitad de las veces)
    - € Casi nunca o nunca

18. **En las últimas 4 semanas, ¿con cuánta frecuencia sintió molestias o dolor después del coito vaginal?**
    - € Sin coito vaginal
    - € Casi siempre o siempre
    - € La mayoría de las veces (más de la mitad de las veces)
    - € A veces (aproximadamente la mitad de las veces)
    - € Pocas veces (menos de la mitad de las veces)
    - € Casi nunca o nunca

19. **En las últimas 4 semanas, ¿cómo valoraría su nivel (grado) de dolor o molestias durante o después del coito vaginal?**
    - € Sin coito vaginal
    - € Muy alto
    - € Alto
    - € Moderado
    - € Bajo
    - € Muy bajo o nada

¡MUCHAS GRACIAS POR SU TIEMPO Y COLABORACIÓN!

www3.uah.es/fisioterapia_saludmujer/

PUNTUACIÓN DEL ÍNDICE DE LA FUNCIÓN SEXUAL FEMENINA

Se suman las puntuaciones del mismo dominio y se multiplica por el factor que corresponde a cada dominio. La puntuación total se obtiene sumando las puntuaciones de los 6 dominios. Una puntuación de 0 en alguno de los dominios indica falta de actividad sexual en el último mes.

DOMINIO	PREGUNTAS	RANGO DE PUNTUACIÓN	FACTOR	PUNTUACIÓN MÍNIMA	PUNTUACIÓN MÁXIMA
**Deseo**	1-2	1-5	0,6	1,2	6
**Excitación**	3-6	0-5	0,3	0	6
**Lubricación**	7-10	0-5	0,3	0	6
**Orgasmo**	11-13	0-5	0,4	0	6
**Satisfacción**	14	0-5	0,4	0,8	6
	15-16	1-5			
**Dolor**	17-19	0-5	0,4	0	6
**RANGO TOTAL**				2	36

PREGUNTA — **PUNTUACIÓN = RESPUESTA**

1. **En las últimas 4 semanas, ¿con qué frecuencia experimentó deseo o interés sexual?**
   5 = Casi siempre o siempre
   4 = La mayoría de las veces (más de la mitad de las veces)
   3 = A veces (aproximadamente la mitad de las veces)
   2 = Pocas veces (menos de la mitad de las veces)
   1 = Casi nunca o nunca

2. **En las últimas 4 semanas, ¿cómo calificaría su nivel (grado) de deseo o interés sexual?**
   5 = Muy alto
   4 = Alto
   3 = Moderado
   2 = Bajo
   1 = Muy bajo o nada

3. **En las últimas 4 semanas, ¿con qué frecuencia sintió excitación sexual durante la actividad sexual o coito vaginal?**
   0 = Sin actividad sexual
   5 = Casi siempre o siempre
   4 = La mayoría de las veces (más de la mitad de las veces)
   3 = A veces (aproximadamente la mitad de las veces)
   2 = Pocas veces (menos de la mitad de las veces)
   1 = Casi nunca o nunca

4. **En las últimas 4 semanas, ¿cómo calificaría su nivel de excitación sexual durante la actividad sexual o coito vaginal?**
   0 = Sin actividad sexual
   5 = Muy alto
   4 = Alto
   3 = Moderado
   2 = Bajo
   1 = Muy bajo o nada

5. **En las últimas 4 semanas, ¿Cuánta confianza tuvo para conseguir excitarse durante la actividad sexual o coito vaginal?**
   0 = Sin actividad sexual
   5 = Confianza muy alta
   4 = Confianza alta
   3 =Confianza moderada
   2 = Confianza baja
   1 = Confianza muy baja o nada

6. En las últimas 4 semanas, ¿con qué frecuencia se sintió satisfecha con su excitación durante la actividad sexual o coito vaginal?

0 = Sin actividad sexual
5 = Casi siempre o siempre
4 = La mayoría de las veces (más de la mitad de las veces)
3 = A veces (aproximadamente la mitad de las veces)
2 = Pocas veces (menos de la mitad de las veces)
1 = Casi nunca o nunca

7. En las últimas 4 semanas, ¿con qué frecuencia consiguió la lubricación vaginal (humedad vaginal) durante la actividad sexual o coito vaginal?

0 = Sin actividad sexual
5 = Casi siempre o siempre
4 = La mayoría de las veces (más de la mitad de las veces)
3 = A veces (aproximadamente la mitad de las veces)
2 = Pocas veces (menos de la mitad de las veces)
1 = Casi nunca o nunca

8. En las últimas 4 semanas, ¿cuánta dificultad encontró para lubricarse (humedad vaginal) durante la actividad sexual o coito vaginal?

0 = Sin actividad sexual
1 = Extremadamente difícil o imposible
2 = Muy difícil
3 = Difícil
4 = Un poco difícil
5 = Sin dificultad

9. En las últimas 4 semanas, ¿con qué frecuencia mantuvo la lubricación vaginal (humedad vaginal) hasta finalizar la actividad sexual o coito vaginal?

0 = Sin actividad sexual
5 = Casi siempre o siempre
4 = La mayoría de las veces (más de la mitad de las veces)
3 = A veces (aproximadamente la mitad de las veces)
2 = Pocas veces (menos de la mitad de las veces)
1 = Casi nunca o nunca

10. En las últimas 4 semanas, ¿cuánta dificultad encontró para mantener la lubricación vaginal (humedad vaginal) hasta finalizar la actividad sexual o coito vaginal?

0 = Sin actividad sexual
1 = Extremadamente difícil o imposible
2 = Muy difícil
3 = Difícil
4 = Un poco difícil
5 = Sin dificultad

11. En las últimas 4 semanas, cuando tuvo una estimulación sexual o coito vaginal, ¿con qué frecuencia consiguió el orgasmo (clímax)?

0 = Sin actividad sexual
5 = Casi siempre o siempre
4 = La mayoría de las veces (más de la mitad de las veces)
3 = A veces (aproximadamente la mitad de las veces)
2 = Pocas veces (menos de la mitad de las veces)
1 = Casi nunca o nunca

12. En las últimas 4 semanas, cuando tuvo una estimulación sexual o coito vaginal, ¿cuánta dificultad tuvo para alcanzar el orgasmo (clímax)?

0 = Sin actividad sexual
1 = Extremadamente difícil o imposible
2 = Muy difícil
3 = Difícil
4 = Un poco difícil
5 = Sin dificultad

13. En las últimas 4 semanas, ¿cuánta satisfacción sintió con su capacidad para alcanzar el orgasmo (clímax) durante la actividad sexual o coito vaginal??

0 = Sin actividad sexual
5 = Muy satisfecha
4 = Moderadamente satisfecha
3 = Ni satisfecha ni insatisfecha
2 = Moderadamente insatisfecha
1 = Muy insatisfecha

14. **En las últimas 4 semanas, ¿cuánta <u>satisfacción</u> sintió con el acercamiento emocional con su pareja durante la actividad sexual?**

0 = Sin actividad sexual
5 = Muy satisfecha
4 = Moderadamente satisfecha
3 = Ni satisfecha ni insatisfecha
2 = Moderadamente insatisfecha
1 = Muy insatisfecha

15. **En las últimas 4 semanas, ¿cuánta <u>satisfacción</u> sintió con la relación sexual con su pareja?**

5 = Muy satisfecha
4 = Moderadamente satisfecha
3 = Ni satisfecha ni insatisfecha
2 = Moderadamente insatisfecha
1 = Muy insatisfecha

16. **En las últimas 4 semanas, ¿cuánta <u>satisfacción</u> sintió con su vida sexual en general?**

5 = Muy satisfecha
4 = Moderadamente satisfecha
3 = Ni satisfecha ni insatisfecha
2 = Moderadamente insatisfecha
1 = Muy insatisfecha

17. **En las últimas 4 semanas, ¿con cuánta <u>frecuencia</u> sintió molestias o dolor <u>durante</u> coito vaginal?**

0 = Sin coito vaginal
1 = Casi siempre o siempre
2 = La mayoría de las veces (más de la mitad de las veces)
3 = A veces (aproximadamente la mitad de las veces)
4 = Pocas veces (menos de la mitad de las veces)
5 = Casi nunca o nunca

18. **En las últimas 4 semanas, ¿con cuánta <u>frecuencia</u> sintió molestias o dolor <u>después</u> del coito vaginal?**

0 = Sin coito vaginal
1 = Casi siempre o siempre
2 = La mayoría de las veces (más de la mitad de las veces)
3 = A veces (aproximadamente la mitad de las veces)
4 = Pocas veces (menos de la mitad de las veces)
5 = Casi nunca o nunca

19. **En las últimas 4 semanas, ¿cómo valoraría su <u>nivel</u> (grado) de dolor o molestias <u>durante o después</u> del coito vaginal?**

0 = Sin coito vaginal
1 = Muy alto
2 = Alto
3 = Moderado
4 = Bajo
5 = Muy bajo o nada
